# Case of Coronavirus Disease 2019 Myocarditis Managed With Biventricular Impella Support

**DOI:** 10.7759/cureus.13197

**Published:** 2021-02-07

**Authors:** Jose G Ruiz, Fadi Kandah, Pooja Dhruva, Maedeh Ganji, Rohan Goswami

**Affiliations:** 1 Cardiology, University of Florida, College of Medicine, Jacksonville, USA; 2 Medicine, University of Florida Health, Jacksonville, USA; 3 Internal Medicine, University of Florida Health, Jacksonville, USA; 4 Cardiology, Mayo Clinic Hospital, Jacksonville, USA

**Keywords:** covid-19, myocarditis, covid and myocarditis, mechanical circulatory support, impella

## Abstract

Severe acute respiratory syndrome coronavirus 2, responsible for coronavirus disease 2019 (COVID-19), is a pandemic that has taken the world by storm. We present the only contemporary reported case of COVID-19 myocarditis leading to recovery with utilization of biventricular Impella (Abiomed, Danvers, MA, USA) for temporary mechanical circulatory support. A 35-year-old female with systemic sclerosis who was found to have five days of generalized malaise associated with fevers and cough. She tested positive for COVID-19 via nasal polymerase chain reaction. Cardiac enzymes were found elevated on admission. Invasive hemodynamics assessment was significant for elevated right and left-sided filling pressures, along with calculated cardiac index of 1.3 L/min/m^2^. Decision was made to place right and left-sided ventricular support with percutaneous Impella for mechanical circulatory support. She was started on intravenous immunoglobulin for suspected COVID-19 myocarditis along with remdesivir and solumedrol. After two weeks of continuous temporary mechanical circulatory support, the patient’s hemodynamics improved and she was discharged. Repeat echocardiogram demonstrated normalization of left ventricular function.

## Introduction

Coronavirus disease 2019 (COVID-19) has changed the healthcare world in a multitude of ways and has forced us to think outside the box in managing patients that are severely affected. Specifically, its various cardiac manifestations, which include myocardial infarctions, myocarditis, and others, have led to profound long-term consequences. Similar to other viruses, COVID-19 has the potential to cause significant myocarditis leading to impaired biventricular (Bi-V) function and chronic heart failure. At its worst, it can result in cardiogenic shock requiring vasopressors and/or inotropes. Longer-term management is still controversial, with temporary mechanical circulatory support growing more popular in these patients. While the exact prevalence of COVID-19 myocarditis is still unclear, the complications that stem from it have raised awareness within the medical community. We present the only contemporary reported case of COVID-19 myocarditis leading to recovery with utilization of Bi-V Impella (Abiomed, Danvers, MA, USA) support for temporary mechanical circulatory support. No cases have been reported regarding utilization of Bi-V Impella as therapy for management of severe acute respiratory syndrome coronavirus 2 (SARS-CoV-2) cardiogenic shock.

## Case presentation

We present the case of a 35-year-old woman with a history of systemic sclerosis who was found to have five days of generalized malaise associated with fevers and cough. On arrival, she was found tachycardic at 112 beats per minute and febrile at 101.8°F. She tested positive for COVID-19 via nasal polymerase chain reaction. Cardiac enzymes were found elevated on admission with troponin T of 0.28. On day two of hospitalization, the patient had spontaneous pulseless electrical activity arrest secondary to hypoxemia from COVID-19 pneumonitis. Transthoracic echocardiogram (TTE) revealed ejection fraction (EF) of less than 10% and severe right ventricular impairment with no pericardial effusion or significant valvular abnormalities seen (Video 1). Previous TTE showed normal LV function.

**Video 1:** TTE: apical four chamber view showing global hypokinesis and EF of 10%. TTE, transthoracic echocardiogram; EF, ejection fraction

**Figure 1:**
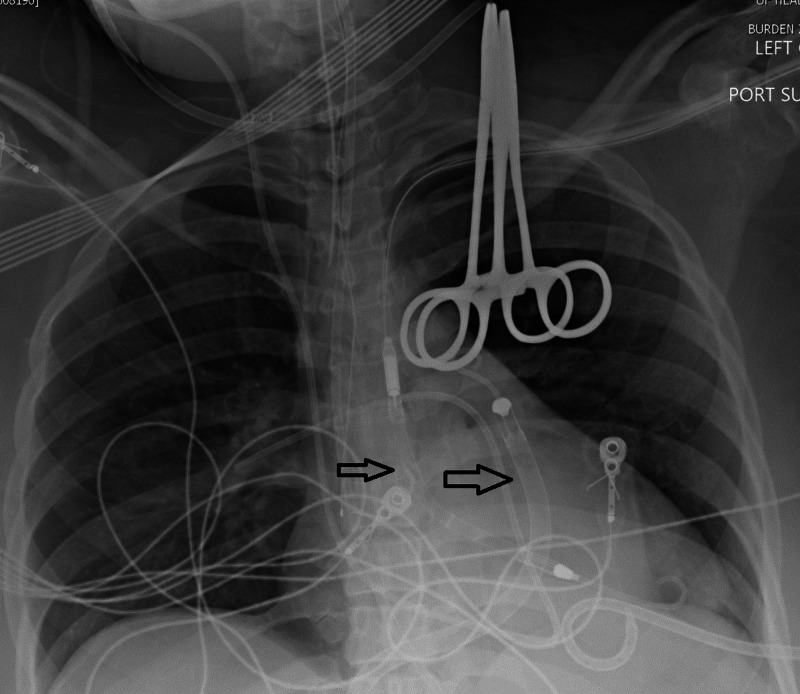
Chest X-ray showing Bi-V Impella placement. Bi-V, biventricular

Labs showed elevated lactic acidosis of 10 and NT pro-BNP of 7139 pg/mL. Invasive hemodynamics demonstrated a right atrial pressure of 21 mmHg, pulmonary arterial pressure of 32/23 (26 mmHg), and pulmonary capillary wedge pressure of 18 mmHg. Pulmonary artery pulsatility index was calculated to be 0.7. CO 2.1 L/min and CI of 1.2 L/min/m^2^ using Fick’s equation. Given the findings consistent with cardiogenic shock, extracorporeal membrane oxygenation (ECMO) was briefly discussed by the surgical team but due to the poor prognosis of the patient and unclear long-term options such as transplant, it was not undertaken. After a heart team approach, the decision was made to place right and left-sided ventricular Impellas’ for mechanical circulatory support (Figure 1).

She was started on intravenous immunoglobulin for COVID-19 myocarditis along with remdesivir and solumedrol. After two weeks of continuous temporary mechanical circulatory support (TMCS), patient hemodynamics improved and she was weaned from TMCS. At this time, cardiac magnetic resonance imaging (MRI) was performed which confirmed the presence of myocarditis. Repeat echocardiogram demonstrated Bi-V recovery and remodeling with an LVEF of 60% and no significant valvular disease or pericardial effusion (Video 2).

**Video 2:** TTE: apical four chamber view showing recovery of EF Post Bi-V Impella. TTE, transthoracic echocardiogram; EF, ejection fraction; Bi-V, biventricular

She was discharge home on day 23 with no neurological deficits.

## Discussion

Myocarditis has been seen in up to 7% of COVID-related deaths [1]. The mechanism behind COVID-induced myocarditis is thought to be due to the combination of direct cell injury through the release of cytokines as well as the virus’s ability to bind to ACE2 spike protein on cardiomyocytes to induce injury [1]. Interestingly, COVID-19 cardiac histopathologies did not demonstrate a high frequency of lymphocyte predominant inflammatory infiltrate with myocyte injury, which is usually seen in viral myocarditis [2]. The most common cardiac findings were nonmyocarditis inflammatory infiltrate, which was reported in about 12.6% of cases [2]. With no distinct histopathology seen to diagnose these patients, about 60% of COVID-19 myocarditis cases were determined through cardiac MRI [2]. As seen in our case, MRI played a role in determining that the patient was suffering from COVID-19 myocarditis.

As a severe form of myocarditis, patients develop signs and symptoms of acute heart failure leading to cardiogenic shock. Initial management includes inotropes, vasopressors, and mechanical ventilation, if needed. However, there is still no consensus on long-term management of these patients. Mechanical circulatory support, which includes either ECMO, ventricular assist device, or intra-aortic balloon pump are some options being used in those unresponsive to conventional therapy [3]. ECMO is not only used in COVID patients with respiratory failure, but is becoming increasingly popular in those with cardiovascular compromise as well. However, there are still no absolute standards on when to initiate ECMO, and data on its effect on overall mortality are still limited.

The use of Bi-V continuous microaxial flow devices during acute COVID-19 myocarditis allows a viable alternative with a minimally invasive approach to allow ventricular rest and optimal offloading without the increased risk of surgically placed TMCS [4]. With recent emergency use status by the FDA, its wide adaptation still remains sparse. Specifically, no cases have been reported regarding utilization of Bi-V Impella as therapy for management of SARS-CoV-2.

## Conclusions

Our case demonstrates a unique approach to management of COVID-19 myocarditis. It is the only reported case in the literature utilizing Bi-V Impella devices for circulatory support without the concurrent use of ECMO. Due to the success in this patient, this promising approach warrants continued investigation in the management of COVID myocarditis and cardiogenic shock.
